# Magazine Notes

**Published:** 1898-09

**Authors:** 


### *MAGAZINE NOTES.*

---

DR. MORITZ BUSCH, who has been sometimes described as Bismarck's Boswell, and who enjoyed terms of special intimacy with the great Chancellor, is the author of an important paper on Bismarck and William I., which will be published entire in *The Living Age* of September 3. It was written with a view to publication after Bismarck's death, and it contains so much that was communicated to the author by Bismarck himself that it is almost autobiographic.

---

THE relations of England and America continue to be much discussed in the English reviews. Two noticeable articles, looking at the question from slightly different points of view, are reprinted in *The Living Age* from *The Nineteenth Century.* One is by Frederick Greenwood and the other by Sir George Sydenham Clarke.

---

PERHAPS the last bit of magazine writing from the pen of Mrs. Oliphant is the article on Siena, contained in *The Living Age* for August 13. It is a charming piece of description.

---

*The Living Age* has lately printed, in its issue for August 6, an article on Social Conditions in America, translated from the leading Italian review, *Nuova Antologia.* The writer, G. M. Flamingo, shows a wider knowledge and a better discrimination than most foreigners treating this subject, and his views are cordial and sympathetic.

